# LOX-1 Deletion Attenuates Myocardial Fibrosis in the Aged Mice, Particularly Those With Hypertension

**DOI:** 10.3389/fcvm.2021.736215

**Published:** 2021-10-12

**Authors:** Xiao Li, Xihe Tang, Bo Liu, Jinghang Zhang, Yongxi Zhang, Hefan Lv, Dongling Liu, Jawahar L. Mehta, Xianwei Wang

**Affiliations:** ^1^Department of Cardiology, The First Affiliated Hospital of Xinxiang Medical University, Xinxiang, China; ^2^Henan Key Laboratory of Medical Tissue Regeneration, Xinxiang Medical University, Xinxiang, China; ^3^Department of Pathology, The First Affiliated Hospital of Xinxiang Medical University, Xinxiang, China; ^4^Department of Oncology, The Third Affiliated Hospital, Xinxiang Medical University, Xinxiang, China; ^5^Division of Cardiology, University of Arkansas for Medical Sciences, Little Rock, AR, United States; ^6^Department of Human Anatomy & Histoembryology, Xinxiang Medical University, Xinxiang, China; ^7^Xinxiang Key Laboratory of Molecular Neurology, Xinxiang Medical University, Xinxiang, China

**Keywords:** myocardial fibrosis, aging, hypertension, collagens, lectin-like oxidized low-density lipoprotein receptor-1 (LOX-1)

## Abstract

**Background:** Lectin-like oxidized low-density lipoprotein receptor-1 (LOX-1) is a transmembrane glycoprotein that mediates uptake of oxidized low-density lipoprotein (ox-LDL) into cells. Previous studies had shown that LOX-1 deletion had a potential to inhibit cardiac fibrosis in mouse models of hypertension and myocardial infarction. Whether LOX-1 deletion also affects cardiac fibrosis associated with aging still remains unknown. The aim of this study was to investigate the effect of LOX-1 deletion on myocardial fibrosis in the aged mice.

**Methods:** C57BL/6 mice and LOX-1 knockout (KO) mice with C57BL/6 background were studied to the age of 60 weeks. Both genotypes of aged mice were exposed to angiotensin II (Ang II) or saline for additional 4 weeks. The mice were then sacrificed, and myocardial fibrosis, reactive oxygen species (ROS) and expression of LOX-1, fibronectin, collagens, p22^phox^, and gp91^phox^ were measured.

**Results:** LOX-1 deletion markedly reduced Ang II-mediated rise of blood pressure in the aged mice (vs. saline-treated mice). LOX-1 deletion also limited fibrosis and decreased fibronectin and collagen-3 expression in the hearts of aged mice, but not the expression of collagen-1 and collagen-4. LOX-1 deletion also inhibited ROS production and p22^phox^ expression. As the aged mice were exposed to Ang II for 4 weeks (resulting in hypertension), LOX-1 deletion more pronounced inhibiting myocardial fibrosis and ROS production, and decreasing expression of fibronectin, collagen-1, collagen-2, collagen-3, p22^phox^, and gp91^phox^.

**Conclusion:** LOX-1 deletion limited fibrosis and ROS production in the hearts of aged mice. This effect was more pronounced in the aged mice with hypertension induced by Ang II infusion.

## Introduction

Heart failure associated with aging is one of the leading causes of death in the world (1). The average age of onset heart failure is 74 years (2). Myocardial fibrosis caused by aging is perhaps the most important cause of heart failure in the elderly (1). With aging, collagens are gradually deposited in myocardial interstitium, resulting in a decrease of elasticity and compliance in ventricles and leading to diastolic heart failure (3). Although many studies have provided different insights into aging-driven myocardial fibrosis, the specific mechanisms are still not completely understood.

Lectin-like oxidized low-density lipoprotein receptor-1 (LOX-1), a 50-KDa type II transmembrane glycoprotein, is an important member in the class E scavenger receptor subfamily (4). The main function of LOX-1 is to recognize and bind oxidized low-density lipoprotein (ox-LDL), and thus mediate the uptake of ox-LDL into cells (5). It is generally acknowledged that LOX-1 is a critical player in atherogenesis (6, 7). Recent studies have implicated LOX-1 in cardiac remodeling following myocardial infarction and in models of hypertension (8–10). Blockade or deletion of LOX-1 has been shown to attenuate myocardial fibrosis in the rodent models (9, 10). However, whether LOX-1 also affects cardiac fibrosis related to aging still remains unknown. Recently, studies from our group and others have indicated that LOX-1 may be involved in aging of cardiovascular system (11–13). LOX-1 was found to be downregulated with aging in vascular walls, endothelial cells, and cardiac fibroblasts (11, 12). An *in vitro* study also showed that LOX-1 downregulation could inhibit proliferation of senescent cardiac fibroblasts by regulating cytoskeleton reorganization (12).

Based on this information, we hypothesized that LOX-1 may participate in myocardial fibrosis associated aging. This study was designed to address this hypothesis by using old wild type (WT) and LOX-1 knockout (KO) mice. Both genotypes of mice were infused with angiotensin II (Ang II) by subcutaneously implanted osmotic pumps to strengthen cardiac fibrosis.

## Methods

### Animal Models

The homozygous LOX-1 KO mice were backcrossed eight times with C57BL/6 strain to replace the genetic background. The genotypes of these mice were verified by genotyping assays. Male LOX-1 KO mice and C57BL/6 WT mice were housed in a conditional room for 56 weeks. Afterwards, both genotypes of mice were randomly divided into four groups (12 animals /group): WT + saline, WT + Ang II, LOX-1 KO + saline, and LOX-1 KO + Ang II. All animals were subcutaneously implanted with osmotic pumps (ALZET® International Distributors, Cupertino, CA, USA) to infuse Ang II (100ng/kg·min for 4 weeks; Abcam, Cambridge, MA, USA) or equal volumes of saline as per previously published protocols (14, 15). Blood pressure was measured every week by noninvasive tail-cuff method with a Softron BP-98A (Softron Co., Ltd., Tokyo, Japan). All experimental procedures were conducted in accordance with protocols approved by the Institutional Animal Care and Usage Committee.

### Hematoxylin-Eosin (HE) and Masson's Trichrome Staining

Hearts were collected from each group of animals (six samples /group). Following washing with PBS twice, the samples were fixed with 4% paraformaldehyde, embedded in paraffin and sliced into 5 μm sections. The ventricle sections were deparaffinized, rehydrated, and stained with HE using standard protocols and with a Masson's Trichrome Stain Kit (Sigma-Aldrich, St. Louis, MO, USA) following manufacturer's instructions. The images were viewed and captured with a digital imaging system. The collagen volume fraction (CVF) was calculated with Image *J* software.

### Immunostaining

Left ventricular sections were blocked with 5% goat serum/1% BSA in PBS for 30 min, and then incubated with LOX-1 antibody (a gift from Dr. Tatsuya Sawamura, Shinshu University, Japan; 1:400, v/v) for 90 min at room temperature. After washing twice with PBS, the sections were incubated with TR-conjugated secondary antibody (ZSGB-Bio, Beijing, China; 1:1,000, v/v) for 30 min at room temperature. After washed with PBS and deionized water, the sections were covered with coverslips by antifade reagent with 4′,6-diamidino-2-phenylindole (DAPI). The images were taken with a fluorescence microscope (Leica Microsystems, Bensheim, Germany). The fluorescence intensity was calculated with Image *J* software.

### DHE Staining

Frozen ventricular tissues were sliced into 7 μm sections, and subsequently the sections were incubated with 5 μM dihydroethidium (DHE) in the dark in a humidified box at 37°C for 30 min. After washing with PBS, the sections were treated with antifade reagent with DAPI and analyzed with a fluorescent microscope. The fluorescence intensity was calculated with Image *J* software.

### Western Blotting

Proteins were extracted from each group of left ventricular tissues with lysis buffer supplemented with protease inhibitor and phenylmethylsulfonyl fluoride (PMSF). Proteins (20 μg /sample) were separated by electrophoresis with 10% SDS/PAGE gels. After electrophoresis, proteins were transferred to nitrocellulose membranes. The blots were blocked with 5% non-fat milk in Tris-buffered saline with Tween-20 (TBS-T) for 1 h at room temperature, and then incubated with primary antibodies against mouse LOX-1 (a gift from Dr. Sawamura; 1:2,000, v/v, dilution), fibronectin (Abcam; 1:2,000, v/v, dilution), collagen-1a (Santa Cruz, Dallas, TX, USA; 1:1,000, v/v, dilution), collagen-3a (Santa Cruz; 1:1,000, v/v, dilution), collagen-4a (Santa Cruz; 1:1,000, v/v, dilution), p22^phox^ (Santa Cruz; 1:1,000, v/v, dilution), gp91^phox^ (Abcam; 1:2,000, v/v, dilution) or β-actin (Santa Cruz; 1:2,000, v/v, dilution) in TBS-T at 4°C overnight on a shaker. After washing with TBS-T three times, the blots were incubated with horseradish peroxidase-conjugated secondary antibodies (Santa Cruz) for 1 h at room temperature. The blots were scanned with a ChemiDOC XRS system (Bio-Rad, Hercules, CA, USA) following exposure to Luminol Reagents (Beyotime, Shanghai, China) for 3 min.

### Statistical Analysis

Statistical analysis was performed with SPSS 15.0 software (IBM, Chicago, IL, USA). Data are presented as mean ± SD from six independent experiments. Univariate comparisons of means were evaluated using one-way ANOVA with Tukey's *post-hoc* adjustment. *P* < 0.05 was considered statistical significance.

## Results

### Confirmation of LOX-1 Expression

**Figure 1:**
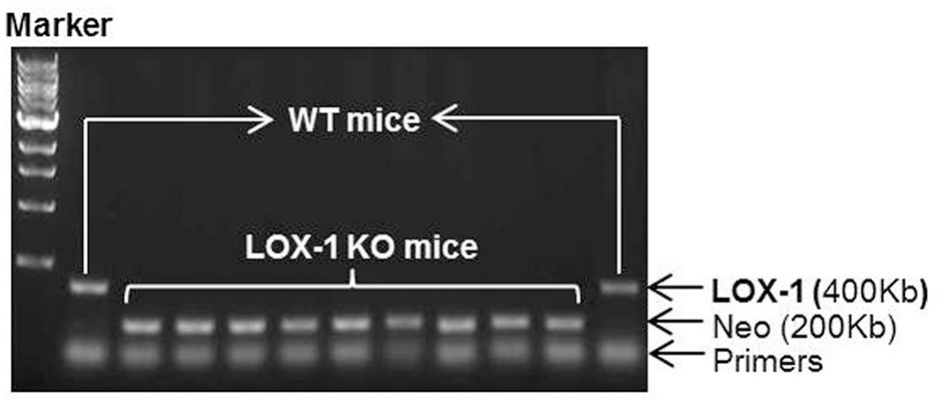
Genotyping results of LOX-1 KO mice and WT mice used in this study. Neo represents the inserted neomycin resistance gene.

**Figure 2:**
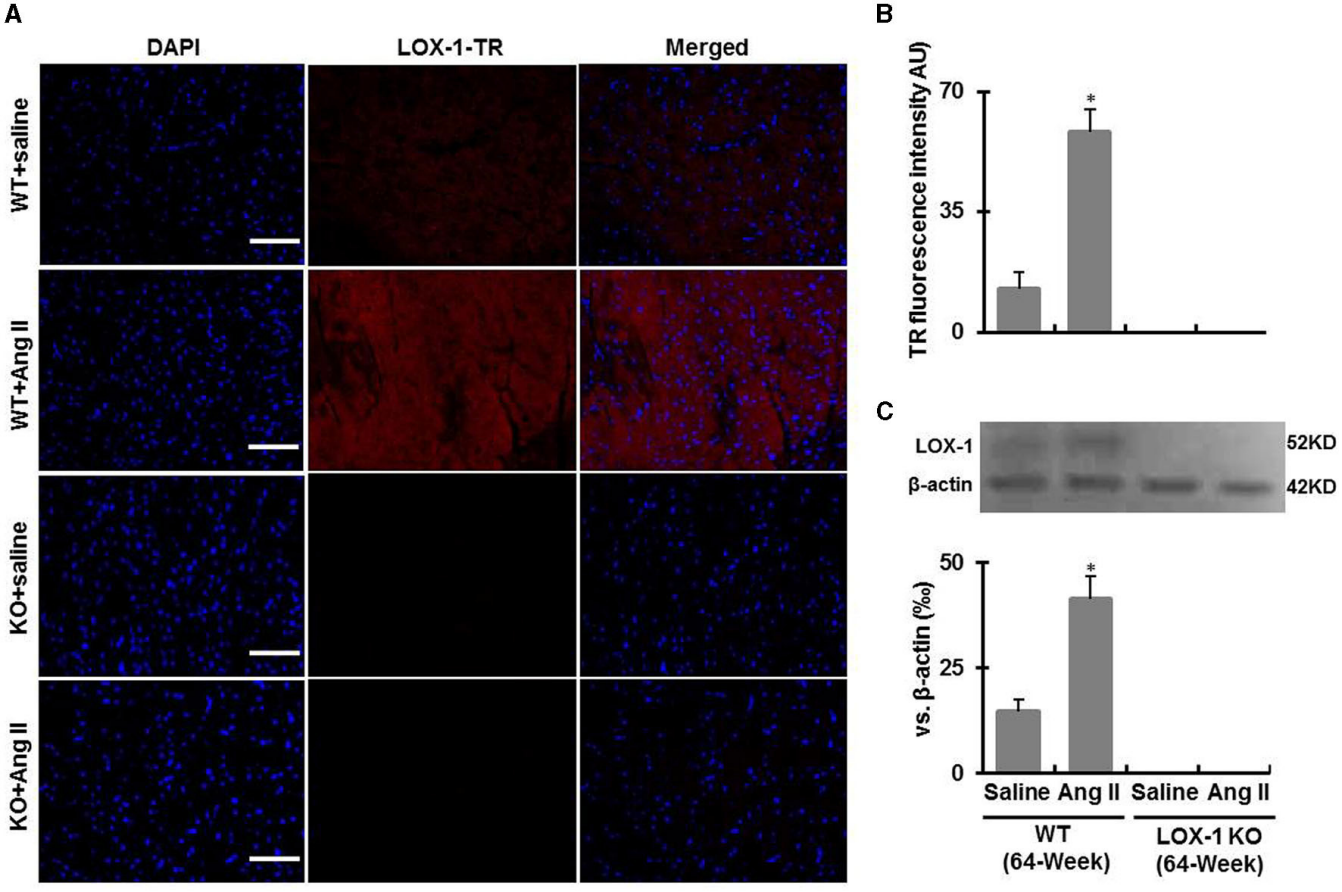
LOX-1 expression in the hearts of each group of mice following infusion with Ang II or saline for 4 weeks. **(A)** Representative immunostaining images show LOX-1 expression in the hearts of each group of mice following Ang II infusion; **(B)** Quantitative data of immunostaining images; **(C)** Western blotting data show LOX-1 expression in the hearts of each group of mice following Ang II infusion. *n* = 6 /group. *P < 0.05, vs. WT + saline group. Scale bars represent 50 μm.

Genotyping data showed that the amplified genomic LOX-1 fragments (400bp) appeared in WT mice following polymerase chain reaction (PCR), but were absent in all LOX-1 KO mice. The band was replaced by inserted neomycin resistance gene fragments (200kb) (Figure 1). Immunofluorescence staining showed that LOX-1 protein expression was increased 4-fold in the hearts derived from WT mice following Ang II infusion for 4 weeks, but was absent in LOX-1 KO mice hearts (Figures 2A,B), which was further confirmed by Western blotting data (Figure 2C).

### LOX-1 Deletion Suppresses Increase of Blood Pressure in Aged Mice With Ang II Infusion

**Figure 3:**
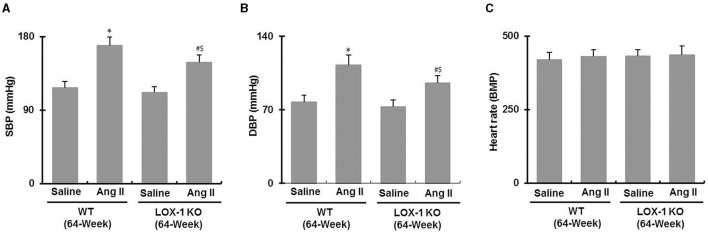
Blood pressure and heart rate in each group of mice following infusion with Ang II or saline for 4 weeks. **(A)** Systolic blood pressure (SBP); **(B)** diastolic blood pressure (DBP); **(C)** heart rate. *n* = 6 /group. *P < 0.05, vs. WT + saline group; ^#^*P* < 0.05, vs. LOX-1 KO + saline group; ^$^*P* < 0.05, vs. WT + Ang II group.

As shown in Figures 3A,B, systolic and diastolic blood pressures both were markedly increased in both WT + Ang II (vs. WT + saline) and LOX-1 KO + Ang II (vs. LOX-1 KO + saline) groups of mice (*P* < 0.05). LOX-1 deletion markedly suppressed Ang II-induced increase of blood pressure in the aged mice (LOX-1 KO + Ang II vs. WT + Ang II, *P* < 0.05), but did not significantly affect blood pressure of aged mice without Ang II infusion (LOX-1 KO + saline vs. WT + saline, *P* > 0.05). Notably, there were no significant differences in heart rate among four groups (*P* > 0.05; Figure 3C).

### LOX-1 Deletion Attenuates Myocardial Fibrosis of Aged Mice Regardless of Infusion With Ang II or Saline

**Figure 4:**
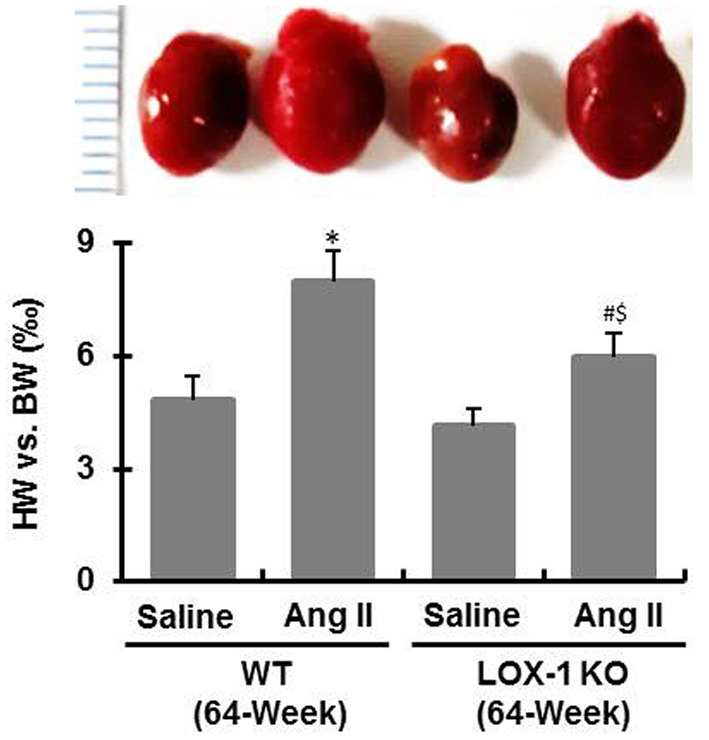
The ratio of heart weight to body weight of each group of mice following infusion with Ang II or saline for 4 weeks. *n* = 6 /group. *P < 0.05, vs. WT + saline group; ^#^*P* < 0.05, vs. LOX-1 KO + saline group; ^$^*P* < 0.05, vs. WT + Ang II group.

**Figure 5:**
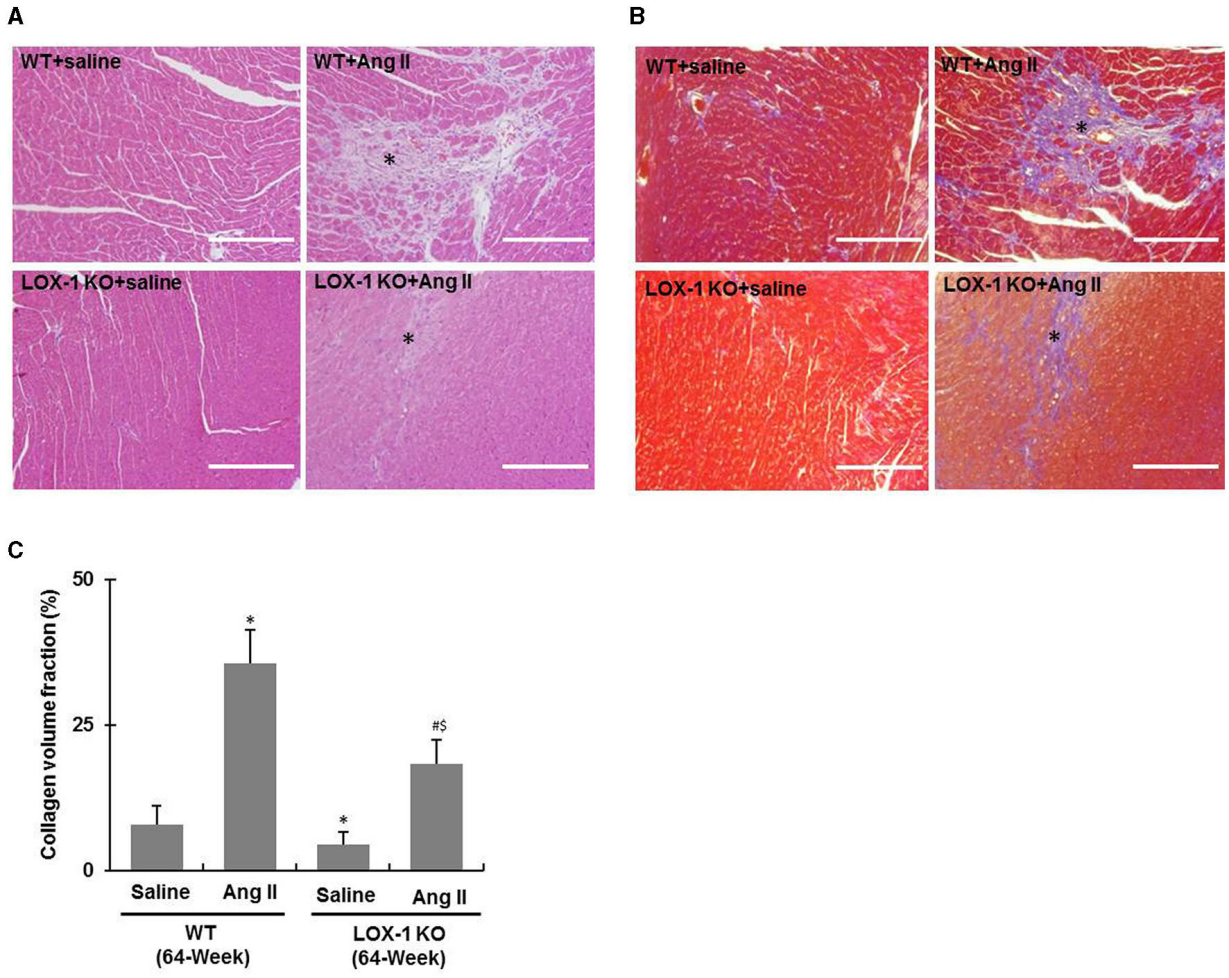
Cardiac fibrosis of each group of mice following infusion with Ang II or saline for 4 weeks. **(A)** Representative HE staining images; **(B)** Representative Masson's trichrome staining images. Asterisks indicating fibrotic scars. **(C)** Quantification of collagen volume fraction in Masson's trichrome staining images. *n* = 6 /group. *P < 0.05, vs. WT + saline group; ^#^*P* < 0.05, vs. LOX-1 KO + saline group; ^$^*P* < 0.05, vs. WT + Ang II group. Scale bars represent 200 μm.

After sacrifice of mice, we measured heart weight and calculated the ratio of heart weight to body weight. As shown in Figure 4, the ratios were markedly increased in WT + Ang II and LOX-1 KO + Ang II groups of mice following infusion with Ang II for 4 weeks, as compared with WT + saline and LOX-1 KO +saline groups, respectively (*P* < 0.05). More importantly, this ratio was markedly smaller in LOX-1 KO + Ang II group than that in WT + saline group (*P* < 0.05), which indicated that LOX-1 deletion could limit cardiac hypertrophy of aged mice with hypertension. Of note, the ratio of heart weight to body weight was also slightly smaller in LOX-1 KO + saline group than that in WT + saline group, but did not reach statistical significance (*P* > 0.05). HE staining data showed that myocytes clearly became hypertrophied in WT + Ang II and LOX-1 KO + Ang II groups as compared with WT + saline and LOX-1 KO +saline groups, respectively (Figure 5A). More importantly, LOX-1 deletion not only limited myocyte hypertrophy in the aged mice with hypertension, but also the aging mice alone (infused with saline) (Figure 5A).

Further, HE staining showed that the fibrotic scar areas were more intense and larger in the hearts of WT + Ang II and LOX-1 KO + Ang II groups of mice as compared with WT + saline and LOX-1 KO +saline groups, respectively (Figure 5A, asterisks indicated). LOX-1 deletion also limited fibrotic scar area in the aged mice with hypertension (Figure 5A, indicated by asterisks). Masson's trichrome staining (Figures 5B,C) further confirmed that LOX-1 deletion could inhibit cardiac fibrosis in the aged mice regardless of infusion with Ang II (aging + hypertension) or saline (aged alone).

**Figure 6:**
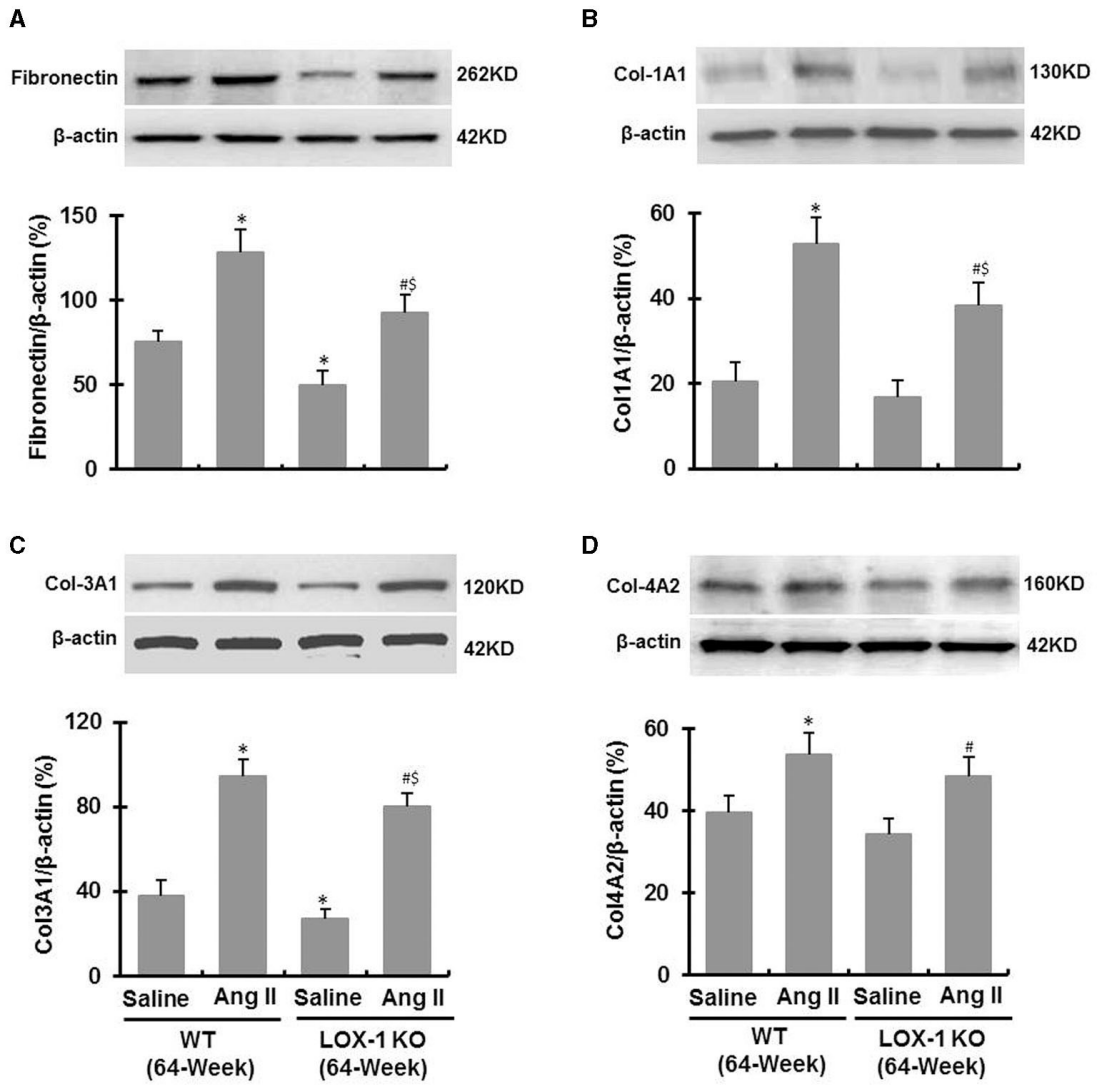
Expression of fibronectin and collagens in the ventricle of each group of mice following infusion with Ang II or saline for 4 weeks (Western blotting). **(A)** Fibronectin expression; **(B)** Collagen-1A1 expression; **(C)** Collagen-3A1 expression; **(D)** Collagen-4A2 expression. *n* = 6 /group. *P < 0.05, vs. WT + saline group; ^#^*P* < 0.05, vs. LOX-1 KO + saline group; ^$^*P* < 0.05, vs. WT + Ang II group.

Western blotting data showed that Ang II infusion significantly increased fibronectin, collagen-1a, collagen-3a and collagen-4 expression in left ventricles of both WT and LOX-1 KO mice (*P* < 0.05; Figures 6A–D), and LOX-1 deletion markedly attenuated Ang II-induced fibronectin, collagen-1 and−3 expression in the hearts of aged mice (*P* < 0.05; Figures 6A–C), but did not significantly affect collagen-4 expression (*P* > 0.05; Figure 6D). In addition, LOX-1 deletion also markedly inhibited fibronectin and collagen-3 expression in the hearts of aged mice infused with saline (*P* < 0.05) (Figures 6A,C), but did not significantly affect collagen-1 and collagen-4 expression (*P* > 0.05) (Figures 6B,D).

### LOX-1 Deletion Inhibits ROS Production in the Hearts of Aged Mice Regardless of Infusion With Ang II or Saline

**Figure 7:**
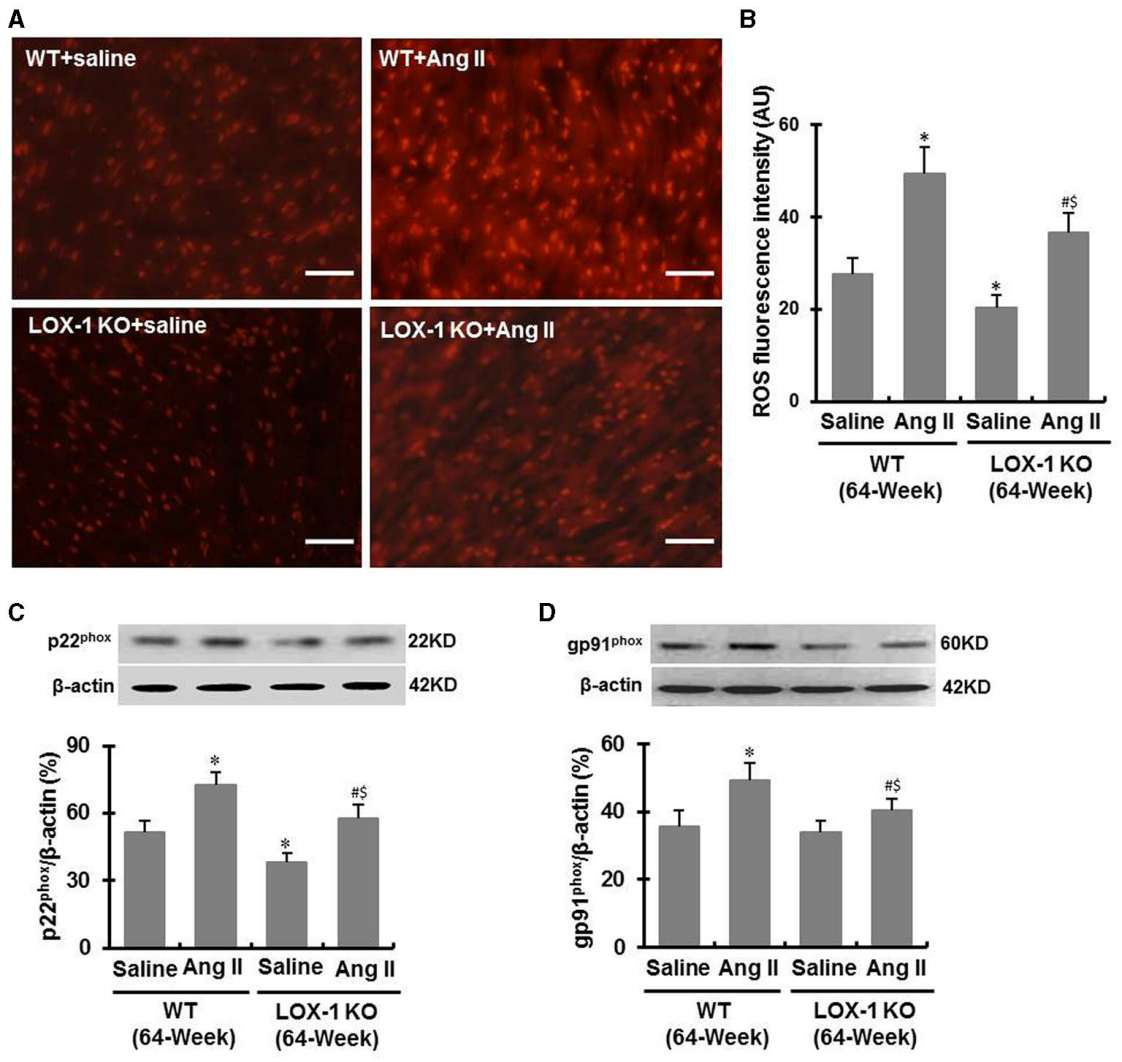
ROS level and expression of p22^phox^ and gp91^phox^ in the ventricle of each group of mice following infusion with Ang II or saline for 4 weeks. **(A)** Representative DHE staining images shows ROS level in the ventricle of each group of mice; **(B)** Quantitative data of DHE staining images; **(C)** Western blotting data show p22^phox^ expression; **(D)** Western blotting data show gp91^phox^ expression. *n* = 6 /group. *P < 0.05, vs. WT + saline group; ^#^*P* < 0.05, vs. LOX-1 KO + saline group; ^$^*P* < 0.05, vs. WT + Ang II group. Scale bars represent 50 μm.

DHE staining showed that ROS levels were markedly increased in the hearts of WT + Ang II and LOX-1 KO + Ang II groups of mice following infusion of Ang II for 4 weeks (*P* < 0.05; Figures 7A,B). LOX-1 deletion attenuated Ang II-induced ROS production in the hearts of aged mice as well as in the hearts aged mice with hypertension (P < 0.05; Figures 7A,B). Western blotting data showed that p22^phox^ and gp91^phox^ expression was also markedly increased in WT + Ang II and LOX-1 KO + Ang II groups of mice following infusion of Ang II for 4 weeks as compared with WT + saline and LOX-1 KO +saline groups, respectively (Figures 7C,D). LOX-1 deletion markedly suppressed p22^phox^ expression in the hearts of aged mice as well as in the hearts of aged mice with hypertension (*P* < 0.05; Figure 7C). Of note, gp91^phox^ expression was suppressed in the aged mice with hypertension (*P* < 0.05), but not the aged mice not given Ang II (*P* > 0.05) (Figure 7D).

## Discussion

Cardiac fibrosis is a chronic process characterized by abnormal collagen accumulation, resulting in myocardium stiffening, reduced contractility, impaired cardiac function and clinical heart failure (16, 17). It is known that aging is an independent risk factor for cardiac fibrosis in the elderly (3). However, the mechanisms of myocardial fibrosis induced by aging needs further studies. LOX-1 has been shown to be involved in myocardial fibrosis following myocardial infarction and sustained hypertension (10, 18). Whether LOX-1 also participate in the aging-induced cardiac fibrosis has still not been elucidated. This is the first study to define the role of LOX-1 in myocardial fibrosis using the natural aging mouse model with or without hypertension. We found that LOX-1 deletion could significantly inhibit myocardial fibrosis and fibronectin and collagen-3 expression in the hearts of aged mice, but not collagen-1 and collagen-4. Furthermore, LOX-1 deletion could also inhibit ROS production and NADPH oxidase subunit p22^phox^ expression in the hearts of aged mice, but not gp91^phox^. In the aged mice with hypertension, LOX-1 deletion attenuated myocardial fibrosis and ROS production, and decreased expression of fibronectin, collagen-1, collagen-2, collagen-3, p22^phox^, and gp91^phox^. These data indicate that LOX-1 deletion has the potential to limit aging-induced myocardial fibrosis, and this process is more pronounced in pathological conditions such as hypertension.

Previous studies indicated that LOX-1 deletion attenuated the evolution of hypertension in young and adult mice or rats presumably by blocking the crosstalk between LOX-1 and renin angiotensin system (RAS) (4, 10, 19). Blockade and deletion of LOX-1 also reduced Ang II type 1 receptor (AT1R) expression in cardiovascular system (18, 20). We postulated that deletion of LOX-1 should reduce evolution of blood pressure in the aged mice. However, in this study we observed that LOX-1 deletion only inhibited blood pressure in the aged mice with Ang II-induced hypertension, but did not affect basal blood pressure of in the aged mice not given Ang II (Figure 3A). It has been reported that there is no significant difference in blood pressure between the aged and young mice (21). Our findings suggest that LOX-1 contributes potency to the evolution of hypertension induced by Ang II, but not to the alterations of blood pressure that occur with aging.

It is known that aging-dependent stimulation to RAS in myocardium induces an increase of NADPH oxidase activity, promotes ROS production, and activates transforming growth factor-β (TGF-β) signaling, which leads to pathological myocardial fibrosis (22, 23). Ang II stimulates collagen synthesis in fibroblasts through activating AT1R (17). In this study, we also observed that cardiac fibrosis and collagen accumulation were markedly enhanced in the hearts of the aged mice given Ang II for 4 weeks (Figures 5, 6). Previous studies demonstrated that Ang II upregulates LOX-1 expression in the heart and other tissues (19, 24). Consistent with previous reports, LOX-1 expression in the hearts of WT aged mice was markedly increased by Ang II infusion, but in the LOX-1 KO mice (Figure 2). LOX-1 deletion inhibited Ang II-induced myocardial fibrosis in the aged mice (Figure 5). LOX-1 deletion also limited myocardial fibrosis of the aged mice infused with saline. These data indicate that LOX-1 deletion has the potential to inhibit cardiac fibrosis of aged mice. This phenomenon is prominent in the aged mice concurrently with Ang II-induced hypertension.

Fibronectin is an important mediator of collagen formation, and collagen-1 and−3 are the major players, and−4 a relatively minor player in matrix accumulation in the hearts under pathological conditions (25). Quantitative protein detection by Western blotting showed that LOX-1 deletion inhibited fibronectin and collagen-3 expression, but not collagen-1 and−4 in the hearts of aged mice infused with saline (Figure 6). Of note, LOX-1 deletion also inhibited different matrix proteins including fibronectin, collagen-1,−3, and−4 in the hearts of aged mice infused with Ang II (Figure 6). This may be the reason why the inhibitory effect of LOX-1 deletion on myocardial fibrosis in the aged mice given Ang II is more prominent than in the aged mice not given Ang II.

ROS are potent stimulators for collagen synthesis and secretion from cardiac fibroblasts and inducers for myocardial fibrosis (26). Aging and LOX-1 expression both cause ROS production in the hearts (18, 27). Our data showed that LOX-1 deletion inhibited ROS production in the hearts of both the aged mice and the aged mice concurrently with hypertension. NADPH oxidative components p22^phox^ and gp91^phox^ are two key regulators of ROS production (28). Previous studies showed that LOX-1 activation influenced NADPH oxidase complex including the subunits p22^phox^, p47^phox^, gp91^phox^, and Rac1, leading to ROS production (5, 29). In this study, we found that LOX-1 deletion suppressed p22^phox^ and gp91^phox^ expression in the hearts of aged mice with hypertension, but only p22^phox^ in the aged mice without hypertension (Figure 7). We suggest that LOX-1 deletion resulting in inhibition of ROS production may partially result from the modulation of p22phox and gp91phox. The effect of LOX-1 deletion on the expression of NADPH oxidative components and ROS was at least partially responsible for more reduction in myocardial fibrosis, especially in the aged mice with hypertension.

In conclusion, we demonstrate for the first time that LOX-1 is an important player in aging-induced cardiac fibrosis, and LOX-1 deletion has the potential to attenuate myocardial fibrosis and inhibit ROS production in the hearts of aging mice. Further, the inhibitory effects LOX-1 deletion on myocardial fibrosis and ROS production appear more prominent in the aged mice that have hypertension. These findings indicate that LOX-1 may be a therapeutic target for the treatment of cardiac fibrosis with aging. Of note, there is a limitation in this study that the extent of myocardial fibrosis was not compared between young and aged mice at baseline and after Ang II infusion.

## Data Availability Statement

The raw data supporting the conclusions of this article will be made available by the authors, without undue reservation.

## Ethics Statement

The animal study was reviewed and approved by Ethics Committee of Xinxiang Medical University.

## Author Contributions

XW: designed the experiments. XL, XT, BL, JZ, YZ, HL, and DL: performed the experiments. XW and JM: wrote the manuscript. All authors contributed to the article and approved the submitted version.

## Funding

This study was funded by the National Natural Science Foundation of China (Nos: 81873459, U1804166, and 81370428 to XW); Henan Outstanding Young Scholars Fund (No: 212300410012 to XW); and the Supporting Plan for Scientific and Technological Innovative Talents in Universities of Henan Province (No: 19HASTIT004 to XW), and the fund from the Department of Veterans Affairs, Veterans Health Administration, Office of Research and Development, Biomedical Laboratory Research and Development, Washington, DC (No: BX000282-09A2 to JM).

## Conflict of Interest

The authors declare that the research was conducted in the absence of any commercial or financial relationships that could be construed as a potential conflict of interest.

## Publisher's Note

All claims expressed in this article are solely those of the authors and do not necessarily represent those of their affiliated organizations, or those of the publisher, the editors and the reviewers. Any product that may be evaluated in this article, or claim that may be made by its manufacturer, is not guaranteed or endorsed by the publisher.
